# Improved repair of dermal wounds in mice lacking microRNA-155

**DOI:** 10.1111/jcmm.12255

**Published:** 2014-03-17

**Authors:** Coen van Solingen, Elisa Araldi, Aranzazu Chamorro-Jorganes, Carlos Fernández-Hernando, Yajaira Suárez

**Affiliations:** a Department of Medicine, Leon H. Charney Division of Cardiology and the Marc and Ruti Bell Vascular Biology and Disease Program, New York University School of MedicineNew York, NY, USA

**Keywords:** microRNAs, microRNA-155^−/−^ mice, wound healing, macrophages, Fizz-1, collagens

## Abstract

Wound healing is a well-regulated but complex process that involves haemostasis, inflammation, proliferation and maturation. Recent reports suggest that microRNAs (miRs) play important roles in dermal wound healing. In fact, miR deregulation has been linked with impaired wound repair. miR-155 has been shown to be induced by inflammatory mediators and plays a central regulatory role in immune responses. We have investigated the potential role of miR-155 in wound healing. By creating punch wounds in the skin of mice, we found an increased expression of miR-155 in wound tissue when compared with healthy skin. Interestingly, analysis of wounds of mice lacking the expression of miR-155 (miR-155^−/−^) revealed an increased wound closure when compared with wild-type animals. Also, the accelerated wound closing correlated with elevated numbers of macrophages in wounded tissue. Gene expression analysis of wounds tissue and macrophages isolated from miR-155^−/−^ mice that were treated with interleukin-4 demonstrated an increased expression of miR-155 targets (BCL6, RhoA and SHIP1) as well as, the finding in inflammatory zone-1 (FIZZ1) gene, when compared with WT mice. Moreover, the up-regulated levels of FIZZ1 in the wound tissue of miR-155^−/−^ mice correlated with an increased deposition of type-1 collagens, a phenomenon known to be beneficial in wound closure. Our data indicate that the absence of miR-155 has beneficial effects in the wound healing process.

## Introduction

Wound healing is a natural restorative response to tissue injury. Upon injury to the skin, a set of complex biochemical events takes place in a closely orchestrated cascade to repair the damage. The wound healing process consists of four highly integrated and overlapping phases: haemostasis, inflammation, proliferation, and maturation [1]. There are many factors that affect wound healing that interfere with one or more phases in this process, causing improper or impaired tissue repair. Immediately after wounding, haemostasis begins, leading to clot formation. The clot and surrounding wound tissue release pro-inflammatory cytokines and growth factors and provides a matrix that attracts and favours the influx of pro-inflammatory cells, namely neutrophils, monocytes/macrophages and lymphocytes [2]. Upon infiltration, the monocytes become activated macrophages that release a variety of cytokines that promote the inflammatory response by recruiting and activating additional leucocytes. In addition to being responsible for clearing apoptotic cells, they are important for initiating the formation of granulation tissue [3]. Adherence to the extracellular matrix (ECM) stimulates monocytes/macrophages to undergo differentiation into a less inflammatory and more reparative state that stimulates the expression of chemoattractants for fibroblasts [3,4]. Fibroblasts, together with macrophages, start to produce and excrete collagens and fibronectin to form a new ECM starting the next, proliferative phase of wound healing, characterized by angiogenesis, wound contraction and re-epithelization [5–7]. Although many cell types participate in the wound healing process, tissue macrophages have been shown to play a critical role, orchestrating the complex processes of inflammation, cellular proliferation and functional tissue regeneration within wounds [8]. In addition, it has been reported that mice depleted of macrophages in early or mid-stages of wound healing have defective wound repair [9,10]. It is therefore clear that macrophages play multiple and pivotal roles in the wound healing process.

It is currently accepted, that small non-coding RNAs, microRNAs (miRs), have the ability to modulate both cell differentiation and cell function [11]. This regulatory control is carried out through repression of gene expression at the post-transcriptional level [12]. Recently, it has been shown that a wide variety of miRs are deregulated upon dermal wounding, suggesting that regulated expression of miRs could influence both wound healing and repair. Indeed, decreased expression of the miR-99 family in keratinocytes supports wound closure [13]. In addition, down-regulation of miR-199-a-5p and miR-200b upon wounding stimulates repair by increasing cutaneous wound angiogenesis [14,15]. In contrast, the expression of miR-210, up-regulated during wound ischaemia, leads to limited keratinocyte proliferation and subsequent impaired wound closure [16]. These examples demonstrate that there is a growing understanding that miRs play a significant role in the various stages of wound healing.

It has become apparent that one specific miR, miR-155, is a central regulator in the immune response [17,18]. The expression of miR-155 is significantly induced in macrophages upon stimulation with inflammatory cytokines [19]. Several reports note severe dysregulation of miR-155 in the pathogenesis of various inflammatory diseases including rheumatoid arthritis and systemic lupus erythematosus [20–23]. In addition, the elevated expression of miR-155 in macrophages within atherosclerotic lesions is demonstrated to be partially responsible for the negative outcome [24].

Considering the clear inflammatory component of wound repair, we hypothesize that miR-155 may play a pivotal role in wound healing. In fact, our data indicate that miR-155 expression is induced in wounds of wild-type (WT) mice. This increase paralleled with elevated numbers of macrophages within the wound area. Interestingly, we found that the deficiency of miR-155 has a beneficial effect on wound closure. Phenotypically, wounds from miR-155^−/−^ mice showed an increased number of infiltrated macrophages compared with WT mice. Interestingly, the expression of the gene found in inflammatory zone (FIZZ1) was increased in the wounds of these animals as was type-1 collagen expression and deposition, a phenomenon known to be beneficial in wound closure [7]. Altogether, these data indicate that miR-155 negatively regulates wound closure and that therapeutic inhibition of miR-155 may be a promising treatment for patients with impaired dermal wound healing.

## Materials and methods

### Animals and wound healing

The Institutional Animal Care Committee of New York University Medical Center approved all animal studies. B6.Cg-Mirn155^tm1.1Rsky^/J (miR-155^−/−^) and C57BL/6J (WT) were obtained from the Jackson Laboratory (Bar Harbor, ME, USA) and maintained on mouse chow and water *ad libitum* at constant temperature and humidity in a 12-h controlled dark/light cycle. Ten- to 12-week-old animals were used for wound healing experiments as previously reported [25]. Briefly, the animals were anesthetized with a ketamine/xylazine solution and two full thickness skin punch biopsies of 6 mm were created using disposable dermal biopsy punches (Acuderm Inc., Ft. Lauderdale, FL, USA). After surgery, the animals were housed in separate cages with *ad libitum* access to chow and water. Ten days post surgery, animals were killed; wounds were excised and bisected. One half was processed for histological analysis, and the other half was snap frozen and stored at −80°C for biochemical analysis.

### Histology and immunohistochemistry

For immunohistochemical analysis, paraffin sections (6 μm) were stained with haematoxylin and eosin, Masson's trichrome (MT), picrosirius red (PSR) or unconjugated antimouse F4/80 rat monoclonal (Life Technologies, Grand Island, NY, USA). Specifically bound primary antibodies were detected with biotinylated anti-rat IgG antibodies (Vector Laboratories, Burlingame, CA, USA), using the Vectastain ABC kit (Vector Laboratories) and combined with 3,3′-Diaminobenzidine (DAB) and Substrate Chromogen System (DAKO, Carpinteria, CA, USA). Images (haematoxylin and eosin, MT, F4/80) were taken with EVOS XL Core Cell Imaging System (Life Technologies); pictures in bright field and with PSR were taken with a microscope suitable for polarized light analysis (Axioplan Wide-Field Fluorescence/DIC upright Microscope; Zeiss, Thornwood, NY, USA). Wound closure was evaluated by measuring total wound area and distance between follicles as reported previously [26]. Total number of macrophages (F4/80-positive) was determined using three randomly assigned wound images per wound. Macrophages were manually counted and subsequently expressed as mean number of cells per high power field. The mean number of three images was used to obtain the mean value per wound/animal.

### Macrophage polarization assay

Bone marrow cells were isolated from the femora and tibiae of WT and miR-155^−/−^ mice and subsequently polarized to an M1 or M2 phenotype as described before [27]. Briefly, after isolation, bone marrow cells were differentiated towards bone marrow–derived macrophages (BMDM) in 20% L929 cell–conditioned media (ATCC, Manassass, VA) in Iscove DMEM (Life Technologies) supplemented with 20% foetal bovine serum (FBS, Life Technologies), penicillin/streptomycin (Invitrogen) and L-glutamine (Life Technologies). After 7 days of differentiation, the BMDMs were polarized for 8 hrs using LPS (Sigma Aldrich, St Louis, MO, USA; 10 ng/ml) and IFNγ IFN- (R&D Systems, Minneapolis, MN, USA; 20 ng/ml) to differentiate towards an M1 phenotype or using IL-4 (R&D Systems; 15 ng/ml) to polarize towards an M2 phenotype. After polarization, cells were harvested for biochemical analysis.

### Quantitative real-time PCR

miR and mRNA levels were quantified as reported previously [28]. Briefly, total RNA was isolated using miRNeasy (Qiagen, Germantown, MD, USA) according to the manufacturer's protocol. For mRNA quantification, cDNA was synthesized using Taqman RT reagents (Life Technologies) following the manufacturer's protocol. Quantitative real-time PCR was performed in triplicate using iQ SYBR green Supermix (Bio-Rad Laboratories, Hercules, CA, USA) on the Eppendorf Mastercycler Realplex (Eppendorf, Hauppauge, NY, USA). mRNA levels were normalized to 18S RNA as an endogenous control for normalization purposes. To specifically detect miRs, the miScript primer assay for miR-155, miR-33 and miR-126 (Qiagen) was used. MiR levels were normalized using snord61.

### Statistical analysis

All data are expressed as means ± SEM. Statistical differences were measured using a one-tailed Student's *t*-test or anova followed by a Bonferroni post-hoc test. A value of *P* < 0.05 was considered statistically significant and shown in graphs with an *, a value of *P* < 0.1 was considered to be a trend towards significance and indicated with an #. Data analysis was performed with GraphPad Prism program (GraphPad Software, La Jolla, CA, USA).

## Results

### Increased levels of miR-155 and macrophages in dermal wounds

**Figure 1:**
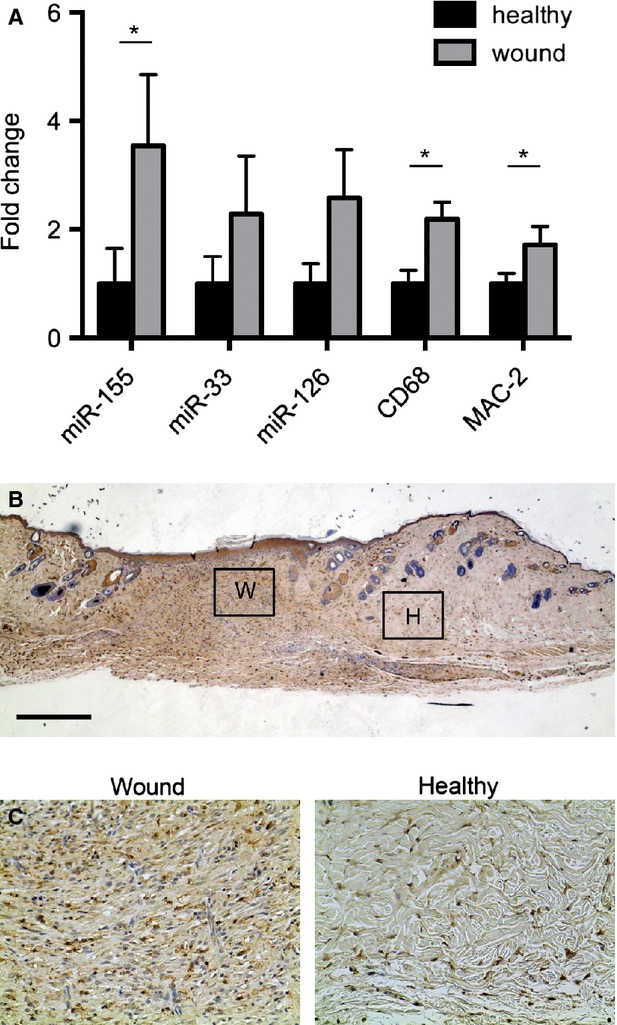
Dermal wounding leads to increased expression of miRs and macrophage markers. (A) Quantitative analysis of miR-155, miR-33 and miR-126 in wound sections showed that expression levels of these miRNAs are up-regulated when compared with healthy skin. qPCR of CD68 and MAC-2 gene expression was used to analyse the influx of macrophages. More macrophages were present in wounded skin when compared with healthy skin. Results are shown as fold difference when compared with mean expression levels of healthy skin using 18S RNA and snord61 as endogenous controls and housekeeping genes for mRNA and miRNA normalization respectively. Results shown are mean ± SEM, *n* = 5 animals, *P < 0.05 with respect to healthy skin using an anova test followed by a Bonferroni post-hoc test. (B) Representative microscopic image of murine skin (wound and healthy) stained for macrophages with F4/80. Bars indicate 500 μm. (C) Areas that are boxed in are zoomed in to show influx of macrophages in wounded (W) and healthy (H) tissue.

Given the role of miR-155 in regulating inflammatory responses and the inflammatory component during wound repair, we analysed expression of miR-155 in healthy skin and compared it with the expression of miR-155 in wounded skin obtained 10 days post wounding. miR-155 levels were significantly higher in the wounded skin (Fig.1A, *P* < 0.05). As expected, the presence of macrophages was increased in wounded tissue, demonstrated by quantitative PCR for the macrophages markers, CD68 and MAC-2 (Fig.1A, *P* < 0.05). The infiltration of macrophages was also revealed in histologic sections of the wounds (Fig.1B and C). To underline the role of miR-155 in wound healing, we analysed the expression of additional miRs. We found that the expression of miR-126, known to be involved in the regulation of angiogenesis and specifically expressed in endothelial cells [29], as well as the expression of miR-33, regulator of cholesterol efflux in macrophages [30], was not significantly altered in wound tissue when compared with healthy skin (Fig.1A, miR-126, *P* < 0.12 miR-33, *P* < 0.20). The trend towards an increased expression of miR-126 and miR-33 could be a consequence of an angiogenic process taking place in the wounds or to an increased macrophage infiltrate, respectively. Altogether, these data indicate that miR-155 is up-regulated after wounding and that this up-regulation could be caused by the infiltration of macrophages in the wounded area.

### Absence of miR-155 leads to better wound closure

**Figure 2:**
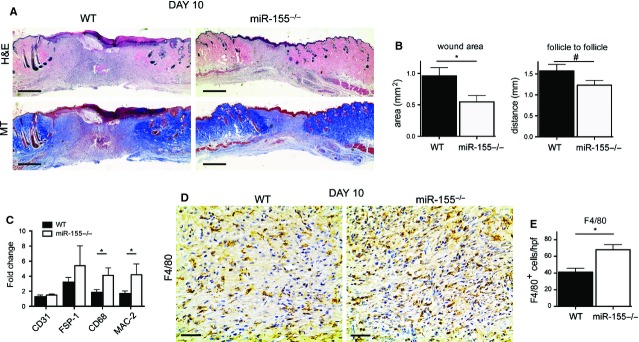
Wounds derived from miR-155^−/−^ mice are smaller 10 days post wounding. (A) Haematoxylin and eosin and Masson's trichome (MT)–stained sections demonstrate decreased area of granulation tissue in wounds derived from miR-155^−/−^ animals when compared with WT. Bars indicate 500 μm. A representative microscopic image out of 10 wounds from 10 animals is shown. (B) Left panel, quantification of total wound area demonstrates a significant decrease in wound area in miR-155^−/−^ compared with WT. Right panel, quantification of the distance between bordering healthy skin, depicted by growth of hair follicle, shows a greater distance in WT animals when compared with miR-155^−/−^. Results shown are mean ± SEM, *n* = 10 per group, *P < 0.05 and #*P* < 0.1 with respect to WT group. (C) Quantitative analysis of the cellular composition of the wound area demonstrated higher expression levels of macrophages makers, CD68 and MAC-1, but not for CD31 and FSP-1, in miR-155^−/−^ group, confirming elevated total numbers of macrophages when compared with WT animals. Results are shown as fold difference when compared with expression levels in healthy skin derived from combining WT and miR-155^−/−^ mice. Results shown are mean ± SEM, *n* = 10 per group, *P < 0.05 with respect to WT group. A representative microscopic image out of 10 stainings form 10 different wounds is shown. (D) Total number of macrophages is elevated in miR-155^−/−^ mice when compared with WT mice. Representative pictures of sections stained with F4/80 in wounded tissue show increased numbers of macrophages (F4/80-positive) in sections derived from miR-155^−/−^ compared with WT. Black bars indicate 50 μm. (E) Quantification of total number of macrophages (F4/80-positive) per high power field (hpf) demonstrates increased numbers of macrophages in miR-155^−/−^ animals.

Next, we investigated the role of miR-155 in dermal wound repair. For this, we performed skin punches in miR-155^−/−^ and control WT mice. Wounds in both groups of animals were examined macroscopically daily for 10 days. Then, the animals were sacrificed, their wounds bisected and used for histological analysis. As demonstrated with haematoxylin and eosin and MT staining of paraffin sections of the wounds. miR-155^−/−^ mice showed a decreased area of granulation tissue when compared with WT animals (Fig.2A). Area quantification confirmed the histological findings and revealed a significantly smaller area of granulation tissue in miR-155^−/−^ (Fig.2B, *P* < 0.05). The formation of *de novo* hair follicles at the edges of a wound is an indication of repaired/healthy skin [31] and therefore this was used as a secondary method to establish wound size. The distance measured between follicles showed a near significant trend between miR-155^−/−^ and WT mice (Fig.2B, *P* < 0.08). Taken together, these data suggest that the absence of miR-155 during wound healing has a positive effect on wound repair.

### Wounds of miR-155^−/−^ mice have an elevated number of macrophages

To investigate the cellular wound composition, we first analysed the expression of specific markers for the main cell types implicated in wound healing, namely CD31, CD68 and fibroblast specific protein-1 (FSP-1), for ECs, macrophages and fibroblasts, respectively. Interestingly, we only found a significant up-regulation of the macrophage marker CD68 in the wound tissue obtained from miR-155^−/−^ mice when compared with WT mice (Fig.2C, *P* < 0.05). This was further confirmed by checking the expression of another macrophage marker, MAC2 (Fig.2C, *P* < 0.05). We then validated the number of macrophages that infiltrated the wound site in miR-155^−/−^ mice compared with WT mice. For this purpose, paraffin sections were stained for the presence of the macrophage marker F4/80 and the total number of positive cells was analysed. As depicted in Figure2D, macrophages were present in the wounds of both WT and miR-155^−/−^ mice. Quantitative evaluation revealed that the number of macrophages was higher in miR-155^−/−^ animals compared with WT mice (Fig.2E, *P* < 0.05). This observation indicates that an elevated number of macrophages is found in the wounds of miR-155^−/−^ animals when compared with WT.

### miR-155 targets and FIZZ1 are up-regulated in macrophages polarized towards an M2 phenotype

**Figure 3:**
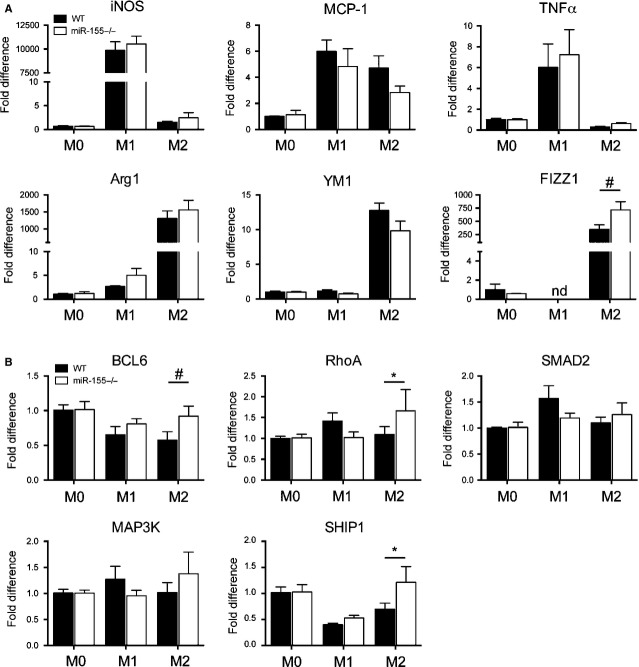
miR-155 targets and FIZZ1 are up-regulated in bone marrow–derived macrophages (BMDMs) after polarization towards M2 phenotype. (A) Quantitative analysis of specific markers for M1 polarization (iNOS, TNF-α and MCP-1) and M2 polarization (Arg-1, YM1 and FIZZ1) in unpolarized BMDM (M0) and BMDMs polarized towards the M1 and M2 phenotypes. M2 macrophages derived from miR-155^−/−^ mice show increased FIZZ1 expression. Graphs show fold change ± SEM of representative results from four independent experiments, #*P* < 0.1 with respect to WT group. (B) Quantitative analysis of validated miR-155 targets show no differences in unpolarized BMDM (M0) or BMDM polarized into an M1 phenotype. M2 phenotype shows up-regulation of the validated miR-155 targets: BCL6, RhoA and SHIP1. Graphs show fold change ± SEM of representative results from four independent experiments, *P < 0.05 and #*P* < 0.1 with respect to WT group.

To examine the role of miR-155 in macrophage function and phenotype, we isolated and cultured BMDMs from WT and miR-155^−/−^ mice. BMDMs were then stimulated with both lipopolysaccharide (LPS) and interferon-gamma (IFN-γ) to differentiate towards an M1 phenotype (classically active macrophages) or with interleukin-4 (IL-4) to polarize towards an M2 phenotype (alternatively activated macrophages)^32^. As a control, vehicle-treated BMDMs were used (M0 phenotype). We found that genes known to be up-regulated after LPS/IFN-γ treatment, such as inducible nitric oxide synthase (iNOS), monocyte chemotactic protein-1 (MCP-1) and tumour necrosis factor-alpha (TNF-α), or after IL-4 treatment, such as Arginase-1 (Arg-1), chitinase 3-like 3 (YM1) and FIZZ1, were induced appropriately in both WT and miR-155^−/−^ BMDMs (Fig.3A), indicating an efficient polarization towards M1 or M2 phenotypes. We then analysed the expression levels of the validated miR-155 targets: B-cell lymphoma 6 protein (BCL6) [24], mitogen-activated protein kinase kinase kinase-10 (MAP3K10) [33], mothers against decapentaplegic homolog-2 (SMAD2) [34], src homology-2 domain-containing inositol 5-phosphatase-1 (SHIP1) [35] and ras homolog gene family member-A (RhoA) [36] in all three macrophage phenotypes. Surprisingly, no differences were found in the expression level of any of these miR-155 gene targets in either M0 or M1 phenotypes when the expression levels of these genes were compared between BMDMs derived from miR-155^−/−^ and WT mice (Fig.3B). In addition, the levels of iNOS, TNF-α and MCP-1, hallmarks of the M1 phenotype [37], were not differentially expressed between the two groups (WT *versus* miR-155^−/−^ BMDMs, Fig.3A). Interestingly, when the expression of miR-155 gene targets was tested in macrophages polarized towards an M2 phenotype (IL-4 treatment), the expression of BCL6, SHIP1 and RhoA was increased in M2 miR155^−/−^ BMDMs (Fig.3B, *P < 0.05 and #*P* < 0.1). Moreover, when we analyzed the expression of Arg-1, YM1 and FIZZ1, all linked with an M2 phenotype [38], only the levels of FIZZ1 were elevated in M2 miR-155^−/−^ BMDM when compared with M2 WT BMDMs (Fig.3A, *P* < 0.1). Taken together, these data imply that miR-155 attenuates the expression of its targets and the expression of FIZZ1 when macrophages are polarized to an M2 phenotype and that these targets (and FIZZ1) are not affected in macrophages with an M1 phenotype.

### miR-155 targets and type-1 collagens are up-regulated in mice lacking miR-155

**Figure 4:**
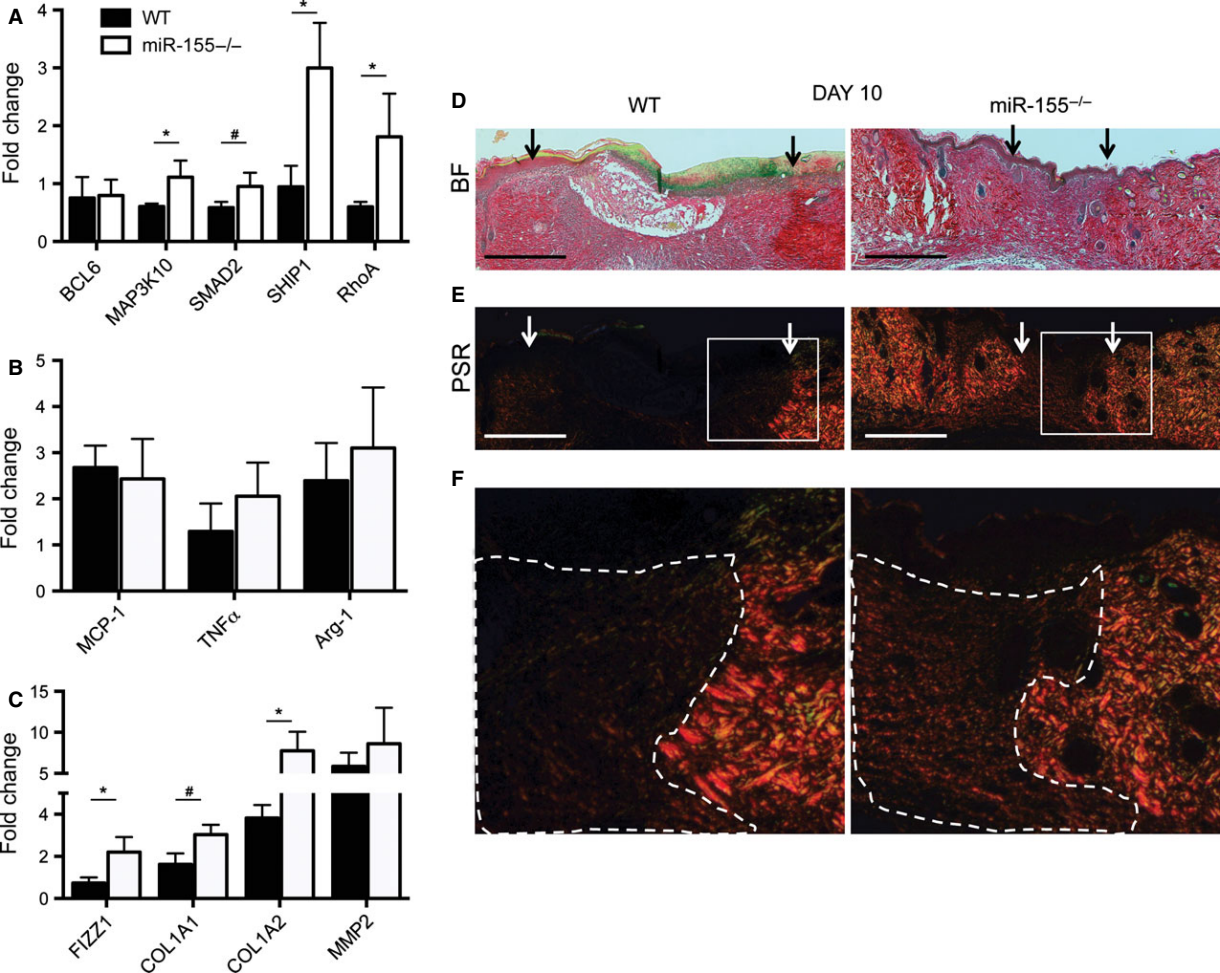
miR-155^−/−^ targets and type-1 collagens are up-regulated in mice lacking the expression of miR-155^−/−^. (A) Quantitative PCR of validated targets of miR-155; BCL6, MAP3K10, SMAD2, SHIP1 and RhoA show differential expression patterns in miR-155^−/−^ mice compared with WT. (B) Quantitative PCR for specific markers of the M1 or M2 macrophage phenotype shows no differences in expression between miR-155^−/−^ mice compared with WT. (C) The expression levels of FIZZ1, COL1A1, Col1A2 are attenuated and MMP2 is unchanged when the two groups were compared. Results are shown as fold difference when compared with expression levels of healthy skin derived from combining WT and miR-155^−/−^ mice. Results shown are mean ± SEM, *n* = 5 out of 10 per group, *P < 0.05 and #*P* < 0.1 with respect to WT, corrected for macrophage numbers using the expression levels of CD68. (D) Bright field pictures of wounds used in Figure2B. (E) Representative wound sections stained for picrosirius red (PSR) shows enhanced staining for type-1 collagens (yellow/orange staining). Borders of wounds are depicted with arrows. Bars indicate 500 μm. (F) Areas that are boxed in are zoomed in to show differences in fluorescence. Dashed area depicts wounded area.

To examine the differences in gene expression in wound tissue of miR-155^−/−^ mice compared with wounds of WT mice, a series of quantitative PCRs were performed. To test whether the absence of miR-155 in wound tissue would attenuate expression levels of validated targets in macrophages, we analysed the gene expression levels of BCL6, MAP3K10, SMAD2, SHIP1 and RhoA (Fig.4A, *P < 0.05 and #*P* < 0.1). Indeed, increased expression of these genes was observed, indicating that the depletion of miR-155 in wound tissue attenuates mRNA expression levels of its targets. In line with the experiments performed with BMDMs, the expression levels of established polarization markers for an M1 phenotype (MCP-1, TNF-α) and an M2 phenotype (Arg-1), corrected for the number of macrophages within the wound, were unaffected in wound tissue of miR-155^−/−^ mice were compared to WT. F and, furthermore, the expression of iNOS and YM1 were totally absent in these wounds. However, in agreement with the results obtained in M2 macrophages, the expression of FIZZ1 (corrected for the number of macrophages) was significantly higher when miR-155^−/−^ wounds were compared with WT (Fig.4C, *P* < 0.05 and Figure S1). Interestingly, FIZZ1 has been described to have a dampening effect on inflammation and also to stimulate the production of type-1 collagens [39]. Therefore, we analysed whether the expression levels of two type-1 collagens, alpha-1 (Col1A1) and alpha-2 (Col1A2), were attenuated by the enhanced expression of FIZZ1. Indeed, we found an increased expression of both genes in the wounds of miR-155^−/−^ (Fig.4C, *P < 0.05 and #*P* < 0.1). To further unravel the role of type-1 collagens and the possible break down of the ECM, the levels of matrix metalloproteinase 2 (MMP2) were tested (Fig.4C). To confirm the quantitative analysis of type-1 collagen expression, we chose to further investigate the deposition of collagens using PSR staining. Indeed, PSR staining confirmed the previously performed quantitative analysis of type-1 collagen expression. Wounds of animals lacking miR-155 showed markedly enhanced staining for type-1 collagens (yellow/orange) when compared with WT mice (Fig.4D–F). Overall, these data illustrate that the expression of miR-155 in wound tissue constricts the levels of FIZZ1 in infiltrating macrophages, thereby influencing the expression and deposition of type-1 collagens during dermal wound repair.

## Discussion

The first stages of dermal wound healing upon injury require a dynamic interplay among leucocytes, monocytes and tissue macrophages. A wide variety of studies have revealed functions of recruited inflammatory cells and resident cells during skin repair [8,40–42]. Recently, the addition of specific roles for miRs in keratinocytes and endothelial cells has extended the complexity of biological processes involved in wound healing [13–16]. Here, we show data on the role of miR-155 during dermal wound repair. Our study shows a marked up-regulation of miR-155 with a simultaneous influx of macrophages in the affected tissue 10 days after inflicting skin wounds on the backs of mice. The influx of macrophages was underlined by the expression of miR-33, a miR associated with macrophages [30].

We demonstrate that the deficiency of miR-155 leads to an enhanced wound closure when compared with WT animals. Surprisingly, this accelerated closure was associated with the presence of an enhanced number of macrophages in the wound tissue. An augmented manifestation of macrophages generally indicates a more inflammatory wound and a possible slower repair [43]. However, the role of the macrophage in wound healing remains incompletely understood as it may well have many functions ranging from the promotion of inflammation to the support of cell proliferation and tissue remodelling [40]. To understand the consequence of the elevated number of macrophages in wounds obtained from mice lacking miR-155, we set out to determine the expression levels of a wide variety of inflammatory genes within the wound tissue. Remarkably, we did not see any difference in the expression levels of established inflammation cytokines TNF-α and MCP-1 between the two groups. Thus, although wounds of miR-155^−/−^ contain more macrophages, the expression of pro-inflammatory markers (M1 phenotype) remains unchanged. This discrepancy implicates a wound environment that might be more skewed towards an anti-inflammatory M2 status. Although debatable, it is recognized that M2 macrophages are depicted to have a reparative function in contrast to their M1-polarized counterparts. M2 macrophages are described as having an anti-inflammatory and pro-angiogenic profile and are considered beneficial during recovery after an inflammatory event [44,45]. FIZZ1 is one of the markers of macrophages that are polarized towards an alternative activated M2 phenotype [46] and increased FIZZ1 expression in wound tissue of miR-155^−/−^ points towards an M2 phenotype and thus a more highly reparative status of the wound tissue. However, another hallmark gene for the M2 phenotype, Arg-1, was not up-regulated in the wounds of miR-155^−/−^ mice when compared with WT mice. This is likely because of the kinetics of expression of this marker in the *in vivo* setting, as its expression was induced *in vitro* in BMDMs from miR155^−/−^ mice treated with IL-4 (M2 phenotype). In fact, most of the effects observed in wound tissue mimicked *in vitro* polarization experiments. The exception was M2 marker, Arg-1, which was consistently slightly increased in LPS/IFN-γ-stimulated macrophages (Fig.3A) derived from miR-155^−/−^ mice. These data are in agreement with the publication of Heymans *et al*. [47]. Overall, like in wound tissue, M1 and M2 macrophages showed no differences in specific markers for either the M1 or M2 phenotype, with the exception of the expression of FIZZ1.

The production of type-1 collagens is mediated by cells migrating into the dermal wound as well as by residential cells 42,48; and this production is detrimental for efficient wound closure [6,49]. Interestingly, it has been described that FIZZ1 can induce the production of type-1 collagens and alpha-smooth muscle actin and thereby directly contributes to ECM formation [22,39]. Indeed, we found enhanced expression of Col1A1 and Col1A2 correlating with the expression of FIZZ1, confirming a beneficial role for FIZZ1 in the production of type-1 collagens and enhanced closure after wounding. Moreover, levels of MMP2 were not changed, indicating that the production of type-1 collagens was not under the influence of a compensatory mechanism driven by MMP2.

The beneficial effect of an increase in endothelial cell function is well studied and well recognized in the process of wound healing [25,50]. Various reports have also described a specific role for miR-155 in endothelial cells and angiogenesis [36,51,52]. However, we did not find any differences in the number of mature endothelial cells 10 days after wounds were created in the skin of the miR-155^−/−^ compared with WT mice (Fig.2 and Figure S2).

In summary, the study presented here shows that miR-155 is up-regulated after infliction of skin wounds in mice and that this correlates to the influx of macrophages into the wound. The wound environment contains (*i*) a more reparative wound milieu as a result of the expression of FIZZ1 and (ii) a higher production of type-1 collagens, all promoting wound repair and contributing to the observed phenotype in miR-155^−/−^ mice with wounds inflicted upon their skin. Altogether, our data indicate that depletion of miR-155 shows clear beneficial effects in wound contraction. Impaired wound healing is a common complication typically seen in patients with type 1 and type 2 diabetes and, despite a clear need, only a few treatments are available that are consistently effective. Counteracting the effects of miR-155 during wound repair could, therefore, herald new venues for targeted therapeutic invention.

## Supporting Information

**Figure S1** Representative microscopic images of wound sections 10 days after surgery.

**Figure S2** Total number of endothelial cells shown no differences in miR-155^−/−^ mice when compared with WT mice.

## References

[1] Gosain A, DiPietro LA (2004). Aging and wound healing. World J Surg.

[2] Martin P, Leibovich SJ (2005). Inflammatory cells during wound repair: the good, the bad and the ugly. Trends Cell Biol.

[3] Singer AJ, Clark RA (1999). Cutaneous wound healing. N Engl J Med.

[4] Brown EJ (1995). Phagocytosis. BioEssays.

[5] Mori R, Shaw TJ, Martin P (2008). Molecular mechanisms linking wound inflammation and fibrosis: knockdown of osteopontin leads to rapid repair and reduced scarring. J Exp Med.

[6] Rodero MP, Legrand JM, Bou-Gharios G (2013). Wound-associated macrophages control collagen 1alpha2 transcription during the early stages of skin wound healing. Exp Dermatol.

[7] Hunt TK, Knighton DR, Thakral KK (1984). Studies on inflammation and wound healing: angiogenesis and collagen synthesis stimulated *in vivo* by resident and activated wound macrophages. Surgery.

[8] Brancato SK, Albina JE (2011). Wound macrophages as key regulators of repair: origin, phenotype, and function. Am J Pathol.

[9] Lucas T, Waisman A, Ranjan R (2010). Differential roles of macrophages in diverse phases of skin repair. J Immunol.

[10] Leibovich SJ, Ross R (1975). The role of the macrophage in wound repair. A study with hydrocortisone and antimacrophage serum. Am J Pathol.

[11] Baltimore D, Boldin MP, O'Connell RM (2008). MicroRNAs: new regulators of immune cell development and function. Nat Immunol.

[12] Doench JG, Sharp PA (2004). Specificity of microRNA target selection in translational repression. Genes Dev.

[13] Jin Y, Tymen SD, Chen D (2013). MicroRNA-99 family targets AKT/mTOR signaling pathway in dermal wound healing. PLoS ONE.

[14] Chan YC, Roy S, Huang Y (2012). The microRNA miR-199a-5p down-regulation switches on wound angiogenesis by derepressing the v-ets erythroblastosis virus E26 oncogene homolog 1-matrix metalloproteinase-1 pathway. J Biol Chem.

[15] Chan YC, Roy S, Khanna S (2012). Downregulation of endothelial microRNA-200b supports cutaneous wound angiogenesis by desilencing GATA binding protein 2 and vascular endothelial growth factor receptor 2. Arterioscler Thromb Vasc Biol.

[16] Biswas S, Roy S, Banerjee J (2010). Hypoxia inducible microRNA 210 attenuates keratinocyte proliferation and impairs closure in a murine model of ischemic wounds. Proc Natl Acad Sci USA.

[17] Sonkoly E, Stahle M, Pivarcsi A (2008). MicroRNAs and immunity: novel players in the regulation of normal immune function and inflammation. Semin Canc Biol.

[18] Roy S, Sen CK (2011). MiRNA in innate immune responses: novel players in wound inflammation. Physiol Genomics.

[19] O'Connell RM, Taganov KD, Boldin MP (2007). MicroRNA-155 is induced during the macrophage inflammatory response. Proc Natl Acad Sci USA.

[20] Stanczyk J, Pedrioli DM, Brentano F (2008). Altered expression of MicroRNA in synovial fibroblasts and synovial tissue in rheumatoid arthritis. Arthritis Rheum.

[21] Divekar AA, Dubey S, Gangalum PR (2011). Dicer insufficiency and microRNA-155 overexpression in lupus regulatory T cells: an apparent paradox in the setting of an inflammatory milieu. J Immunol.

[22] Junker A, Krumbholz M, Eisele S (2009). MicroRNA profiling of multiple sclerosis lesions identifies modulators of the regulatory protein CD47. Brain.

[23] Takagi T, Naito Y, Mizushima K (2010). Increased expression of microRNA in the inflamed colonic mucosa of patients with active ulcerative colitis. J Gastroenterol Hepatol.

[24] Nazari-Jahantigh M, Wei Y, Noels H (2012). MicroRNA-155 promotes atherosclerosis by repressing Bcl6 in macrophages. J Clin Invest.

[25] Suarez Y, Fernandez-Hernando C, Yu J (2008). Dicer-dependent endothelial microRNAs are necessary for postnatal angiogenesis. Proc Natl Acad Sci USA.

[26] Eming SA, Werner S, Bugnon P (2007). Accelerated wound closure in mice deficient for interleukin-10. Am J Pathol.

[27] Pelegrin P, Surprenant A (2009). Dynamics of macrophage polarization reveal new mechanism to inhibit IL-1beta release through pyrophosphates. EMBO J.

[28] Chamorro-Jorganes A, Araldi E, Penalva LO (2011). MicroRNA-16 and microRNA-424 regulate cell-autonomous angiogenic functions in endothelial cells *via* targeting vascular endothelial growth factor receptor-2 and fibroblast growth factor receptor-1. Arterioscler Thromb Vasc Biol.

[29] van Solingen C, Seghers L, Bijkerk R (2009). Antagomir-mediated silencing of endothelial cell specific microRNA-126 impairs ischemia-induced angiogenesis. J Cell Mol Med.

[30] Rayner KJ, Suarez Y, Davalos A (2010). MiR-33 contributes to the regulation of cholesterol homeostasis. Science.

[31] Ito M, Yang Z, Andl T (2007). Wnt-dependent *de novo* hair follicle regeneration in adult mouse skin after wounding. Nature.

[32] Gordon S (2003). Alternative activation of macrophages. Nat Rev Imminol.

[33] Zhu J, Chen T, Yang L (2012). Regulation of microRNA-155 in atherosclerotic inflammatory responses by targeting MAP3K10. PLoS ONE.

[34] Louafi F, Martinez-Nunez RT, Sanchez-Elsner T (2010). MicroRNA-155 targets SMAD2 and modulates the response of macrophages to transforming growth factor-{beta}. J Biol Chem.

[35] O'Connell RM, Chaudhuri AA, Rao DS (2009). Inositol phosphatase SHIP1 is a primary target of miR-155. Proc Natl Acad Sci USA.

[36] Bijkerk R, de Bruin RG, van Solingen C (2012). MicroRNA-155 functions as a negative regulator of RhoA signaling in TGF-β-induced endothelial to mesenchymal transition. MicroRNA.

[37] Martinez FO, Sica A, Mantovani A (2008). Macrophage activation and polarization. Front Biosci.

[38] Martinez FO, Helming L, Gordon S (2009). Alternative activation of macrophages: an immunologic functional perspective. Annu Rev Immunol.

[39] Liu T, Dhanasekaran SM, Jin H (2004). FIZZ1 stimulation of myofibroblast differentiation. Am J Pathol.

[40] Koh TJ, DiPietro LA (2011). Inflammation and wound healing: the role of the macrophage. Expert Rev Mol Med.

[41] Barisic-Dujmovic T, Boban I, Clark SH (2010). Fibroblasts/myofibroblasts that participate in cutaneous wound healing are not derived from circulating progenitor cells. J Cell Physiol.

[42] Higashiyama R, Nakao S, Shibusawa Y (2011). Differential contribution of dermal resident and bone marrow-derived cells to collagen production during wound healing and fibrogenesis in mice. J Invest Dermatol.

[43] Diegelmann RF, Evans MC (2004). Wound healing: an overview of acute, fibrotic and delayed healing. Front Biosci.

[44] Goerdt S, Politz O, Schledzewski K (1999). Alternative *versus* classical activation of macrophages. Pathobiology.

[45] Mosser DM, Edwards JP (2008). Exploring the full spectrum of macrophage activation. Nat Rev Immunol.

[46] Raes G, De Baetselier P, Noel W (2002). Differential expression of FIZZ1 and Ym1 in alternatively *versus* classically activated macrophages. J Leukoc Biol.

[47] Heymans S, Corsten MF, Verhesen W (2013). Macrophage microRNA-155 promotes cardiac hyprtrophy and failure. Circulation.

[48] Fathke C, Wilson L, Hutter J (2004). Contribution of bone marrow-derived cells to skin: collagen deposition and wound repair. Stem Cells.

[49] Witte MB, Barbul A (1997). General principles of wound healing. Surg Clin North Am.

[50] Wietecha MS, Chen L, Ranzer MJ (2011). Sprouty2 downregulates angiogenesis during mouse skin wound healing. Am J Physiol.

[51] Kong W, He L, Richards EJ (2014). Upregulation of miRNA-155 promotes tumour angiogenesis by targeting VHL and is associated with poor prognosis and triple-negative breast cancer. Oncogene.

[52] Liu T, Shen D, Xing S (2013). Attenuation of exogenous angiotensin II stress-induced damage and apoptosis in human vascular endothelial cells *via* microRNA-155 expression. Int J Mol Med.

