# Supplementary figures and images for: Improved repair of dermal wounds in mice lacking microRNA-155
Source: J Cell Mol Med. 2014 Mar 17;18(6):1104–12. doi: 10.1111/jcmm.12255 (PMC4112003; doi:10.1111/jcmm.12255)

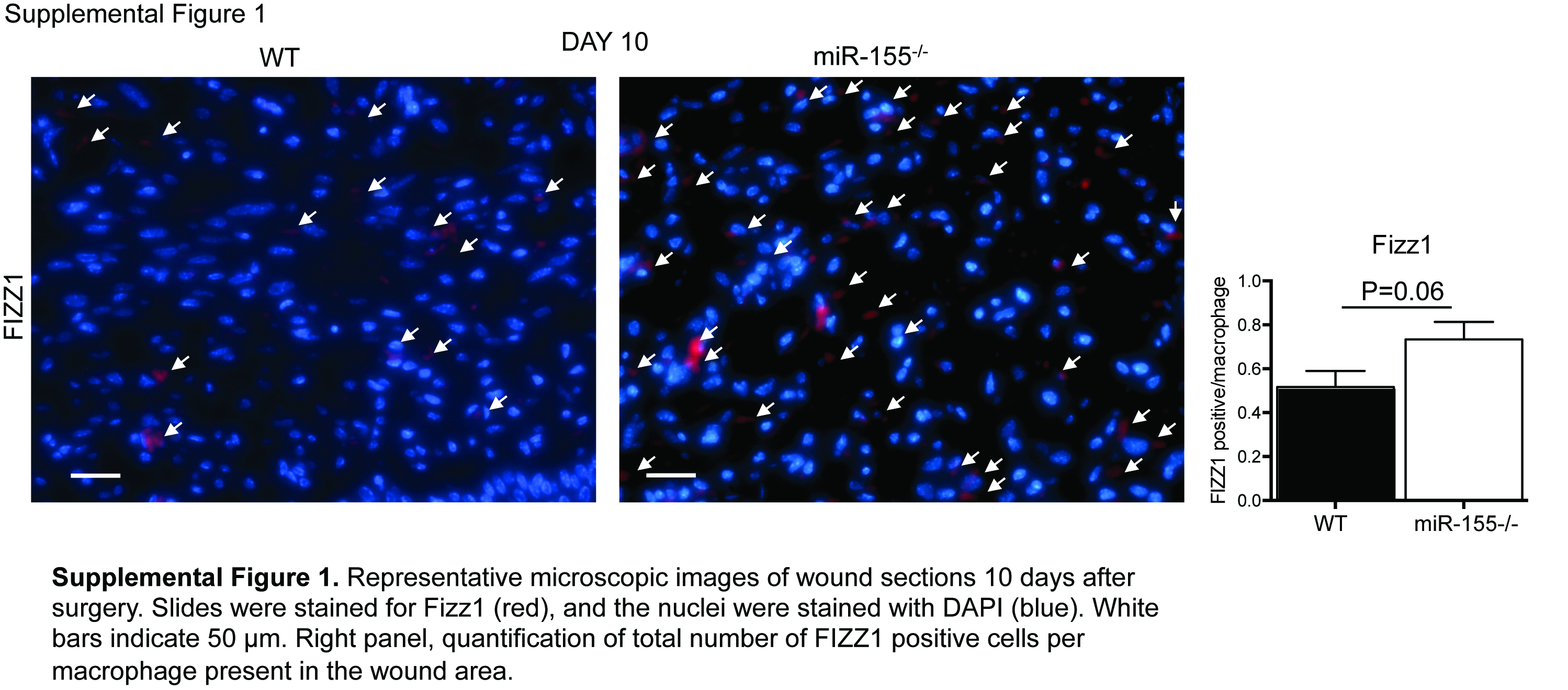

Supplement: Supplementary file 1 [file jcmm0018-1104-sd1.tif]

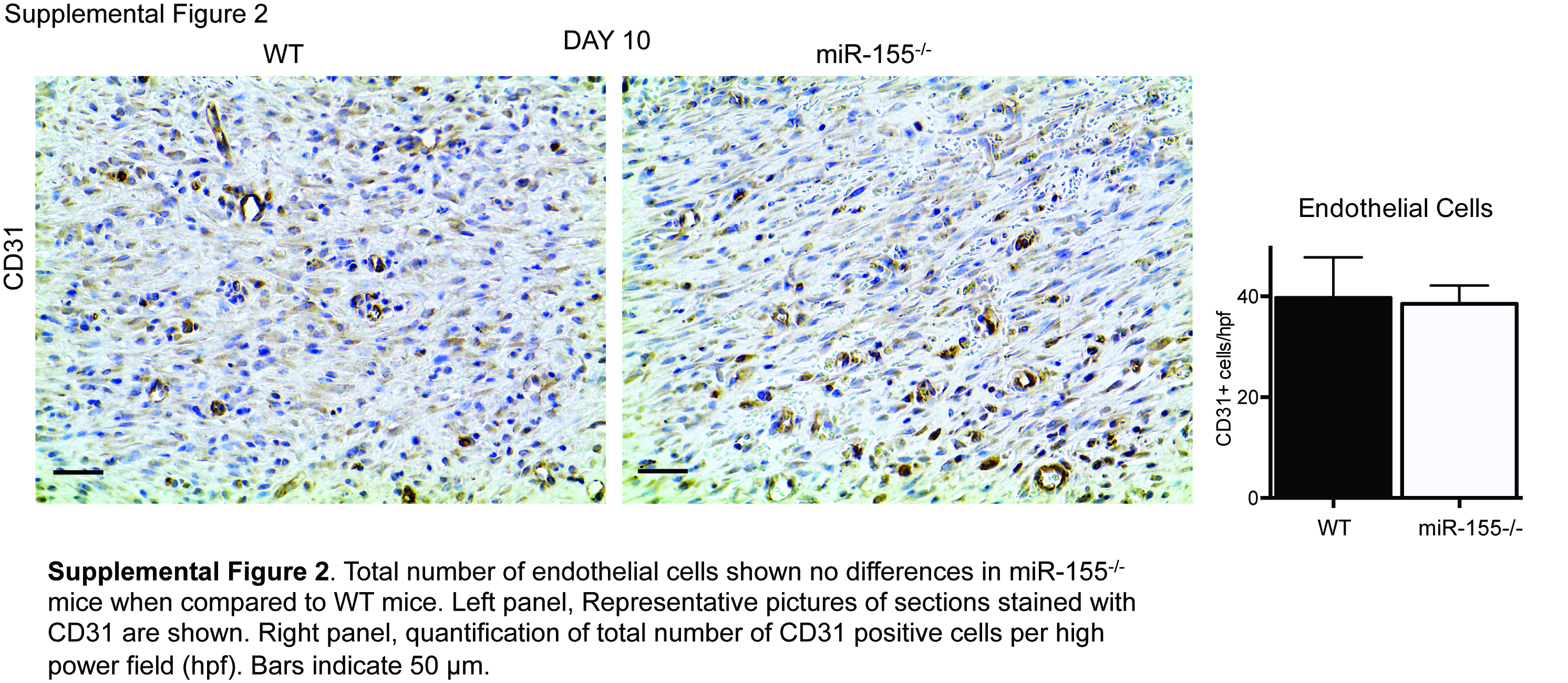

Supplement: Supplementary file 2 [file jcmm0018-1104-sd2.tif]
