# Supplementary material for: Improved repair of dermal wounds in mice lacking microRNA-155
Source: J Cell Mol Med. 2014 Mar 17;18(6):1104–12. doi: 10.1111/jcmm.12255 (PMC4112003; doi:10.1111/jcmm.12255)
Supplement: Supplementary file 3 [file jcmm0018-1104-sd3.docx]

**Supplemental Information**

**Material and Methods**

**Immunohistochemistry**

For immunohistochemical analysis paraffin sections (6 μm) were stained with unconjugated rabbit anti-mouse CD31 (Abcam, Cambridge, Ma) and rabbit anti-mouse FIZZ1 (anti-RELMα, Abcam). Specifically bound primary CD31- antibodies were detected with biotinylated anti-rabbit IgG antibodies (Vector Laboratories, Burlingame, CA), using the Vectastain ABC kit (Vector Laboratories) and combined with 3,3’-Diaminobenzidine (DAB) and Substrate Chromogen System (DAKO, Carpinteria, CA). Specifically bound Fizz-1 was detected and visualized with anti rabbit IgG antibodies with alexafluor-568 (Life Technologies, Molecular Probes, Grand Island, NY). Images were taken with EVOS XL Core Cell Imaging System (Life Technologies. Total number of endothelial cells (CD31-postive) and FIZZ1 positive cells were determined using three randomly assigned wound images per wound, manually counted and subsequently expressed as mean number of cells per high power field.
